# Preoperative anaemia and perioperative red blood cell transfusion as prognostic factors for recurrence and mortality in colorectal cancer—a Swedish cohort study

**DOI:** 10.1007/s00384-016-2678-3

**Published:** 2016-10-21

**Authors:** Malin E. M. Mörner, Gustaf Edgren, Anna Martling, Ulf Gunnarsson, Monika Egenvall

**Affiliations:** 1 Department of Clinical Science Intervention and Technology, Karolinska Institutet, Stockholm, Sweden; 2 Functional Area of Emergency Medicine Huddinge, C1:63, Karolinska University Hospital, Huddinge, 141 86 Stockholm, Sweden; 3 Department of Medical Epidemiology and Biostatistics, Karolinska Institutet, Stockholm, Sweden; 4 Department of Molecular Medicine and Surgery, Karolinska Institutet, Stockholm, Sweden; 5 Center of Digestive Diseases, P9:03, Karolinska University Hospital, Solna, Sweden; 6 Department of Surgical and Perioperative Sciences, Umeå university, Umeå, Sweden

**Keywords:** Anaemia, Transfusion, Colorectal cancer, Recurrence, Mortality, Survival

## Abstract

**Purpose:**

The hypothesis in this study was that anaemia prior to surgery and perioperative red blood cell transfusion increases the risk for recurrence and overall mortality in patients with stages I–III colorectal cancer after abdominal resection with curative intent.

**Methods:**

This is a Swedish single centre retrospective cohort study. Data on 496 consecutive radical abdominal resections stages I–III colorectal cancer performed at the Karolinska University Hospital 2007–2010 were extracted from the Swedish Colorectal Cancer Registry. Data were linked to local laboratory and transfusion databases to identify preoperative anaemia and perioperative transfusion. Disease recurrence was validated by scrutiny of patient records. A total of 496 stages I–III colorectal cancer patients were included in the analysis. Multivariate Cox regression analysis adjusted for tumour and patient characteristics were performed to assess risk for recurrence and overall mortality.

**Results:**

Anaemia prior to surgery was associated with increased risk for overall mortality (HR 2.1, 95% CI 1.4–3.2). There was no association between anaemia and risk for recurrence (HR 1.6, 95% CI 0.97–2.6). Transfusion was not associated with increased risk of recurrence (HR 0.7, 95% CI 0.4–1.3) or overall mortality (HR 1.04, 95% CI 0.7–1.6).

**Conclusions:**

Anaemia prior to colorectal cancer surgery was associated with increased risk for overall mortality while a no increased risk was seen for recurrence. Previous findings indicating an association between blood transfusion and increased risk for recurrence could not be confirmed.

## Introduction

Anaemia is common in colorectal cancer [1–3] (CRC), with a reported prevalence ranging from 23 to 75 % [1–4]. Worldwide, CRC is the third most common cancer in men and second most common in women [5]. Iron deficiency anaemia in CRC thus constitutes an important clinical problem with no clear guidelines for management. Anaemia in CRC patients is mainly caused by bleeding from the tumour; either occultly from a proximal colon tumour [2, 6], or with visible faecal blood from distal colon tumours or rectal cancers [2, 7]. However, anaemia can also be caused by a systemic inflammatory response to the tumour [8].

Iron deficiency anaemia can be corrected by blood transfusion or by the administration of iron medication prior to surgery [9]. Perioperative blood transfusion is considered to be associated with an increased risk for recurrence in CRC patients operated with curative intent [10]. This dose-dependent negative effect was independent of stage or when, during the perioperative period, the transfusion was administered [10].

The importance of anaemia in patients with CRC has only been investigated in a few studies. Anaemia has been associated with more advanced stage [11] and higher mortality [11, 12]. In another study on patients with T3N0M0 and T4N0M0 colon cancer, impaired disease-free survival associated with anaemia was only seen in the former group [13].

The hypothesis tested in this study was that preoperative anaemia and perioperative red blood cell transfusion, in patients with localised CRC undergoing curative resection, increase the risk for recurrence and overall mortality.

## Materials and methods

### Data sources

This is a single centre, retrospective cohort study, on resections for CRC stages I–III performed at the Karolinska University Hospital 2007–2010. Data on all consecutive patients living in the region were retrieved from the Swedish Colorectal Cancer Registry (SCRCR) [14, 15] on 7 January 2014. Data were not retrieved on patients referred from other regions since they are followed up where they live. The SCRCR, including prospectively collected data, contains information on the patient, neo-adjuvant treatment, surgical procedure, tumour characteristics, and short- and long-term follow-up. Information on all red cell concentrate transfusions was obtained from the local blood transfusion database [16], and on all preoperative haemoglobin concentrations from the local laboratory database. For patients with no available records in the laboratory database (*n* = 21), data on preoperative haemoglobin concentrations were obtained through review of case records. Information on recurrence was validated by scrutiny of patient records and entered into a common database. Record linkage was performed using the patient’s unique national registration number assigned to all Swedish citizens upon birth or immigration [17]. After local processing, records from the SCRCR, transfusion and laboratory databases were submitted to the Swedish National Board of Health and Welfare for further record linkage with data not used in this study and anonymisation of the database.

Only resections judged as locally radical and curative by the surgeon and radical by the pathologist (R0) were included (leaving 546 patients). Patients previously operated for CRC (*n* = 12) or operated for metachronous CRCs (*n* = 16) during the study period were excluded. Patients dying during the initial 30 days after surgery or within the same hospital stay if longer (*n* = 14) and patients with missing haemoglobin values (*n* = 8) were also excluded leaving 496 patients for the final analysis: 282 colon cancers and 214 rectal cancers.

Main outcomes were recurrence and overall death.

Anaemia was defined according to the WHO classification of anaemia for the adult population (age ≥ 15 years); a haemoglobin less than 120 g/l in non-pregnant women and a haemoglobin less than 130 g/l in men [18] within 2 months before the date of surgery. Transfusion was defined as having received at least one allogeneic red blood cell transfusion within 1 day of surgery. Analyses were also performed for patients receiving a transfusion within 30 days of surgery. Operations were classified and divided into three groups: colon resection, low anterior resection and Hartmann’s procedure (for rectal cancer) and a third group with abdomino-perineal resection.

A local recurrence or metastases diagnosed by radiology or histopathology and verified by viewing the patient records was defined as a recurrence.

### Statistical analysis

Categorical variables were described as frequency and percentages. Age was described as median with interquartile range (IQR).

Differences in the distribution of gender, tumour site, ASA class, pT Stage and pN stage between patients with and without anaemia were analysed using the chi-square test. Multivariate adjusted analyses were performed using the Cox Proportional Hazards model for calculation of Hazard ratios. The multivariate analyses were adjusted for parameters suspected of being associated both with anaemia/transfusion and risk for recurrence and overall mortality; gender, ASA class, pT Stage, pN Stage, type of surgery and neo-adjuvant treatment, all as categorical variables, and for age and blood loss as restricted cubic splines with three degrees of freedom [19, 20]. This made it possible to adjust for age and blood loss as functions of continuous variables.

In the analysis of risk for recurrence, patients were followed from the date of surgery until the date of first recurrence, death or end of follow-up (2014-01-07), whichever came first. In the analysis of overall mortality, patients were followed from the date of surgery until date of death, or end of follow-up (2014-01-07), whichever came first. Data are presented as hazard ratios (HR) with 95 % confidence intervals (CI). Relative risk is used in the text as being equivalent to hazard ratio.

To assess the appropriateness of including both anaemia and transfusion in the same regression model, co-linearity between these variables was tested using the chi-square test. This analysis revealed some evidence of moderately strong co-linearity, and we therefore conducted separate analyses and analyses with both variables in the same model.

Kaplan-Meyer curves showing risk for recurrence and overall survival were compared with log-rank test to test for significant differences between groups.

A *p* value <0.05 was considered statistically significant. STATA/IC 11.2 was used for statistical analyses and figures.

## Results

Patient characteristics according to grade of anaemia and blood transfusion are given in Tables 1 and 2. Of the anaemic patients, 150 (58 %) received a transfusion within 24 h of surgery compared to 49 (21 %) of the non-anaemic patients. In total, 199 patients in the cohort received a red cell blood transfusion within 24 h of their resection. In addition, 46 patients received transfusion more than 24 h before or after surgery within 30 days before or after surgery.

**Table 1:** Patient characteristics of 496 patients undergoing curative abdominal resections for stage I–III colorectal cancer

Patient characteristics	No anaemia	Anaemia
Subjects, *N* (% of total)	239 (48.2)	257 (51.8)
Female, *N* (%)	115 (48.1)	119 (46.3)
Median age (year) at diagnosis^a^ (IQR)	67 (61–74)	70 (62–79)
Number CRC, no. (%)		
Colon	115 (48.1)	167 (65.0)
Rectum	124 (51.9)	90 (35.0)
ASA, *N* (%)		
1	50 (20.9)	30 (11.7)
2	132 (55.2)	113 (44.0)
3	48 (20.1)	100 (38.9)
4	9 (3.8)	14 (5.4)
pT stage, *N* (%)		
1	43 (18.0)	14 (5.4)
2	66 (27.6)	27 (10.5)
3	109 (45.6)	154 (59.9)
4	21 (8.8)	62 (24.1)
pN stage, *N* (%)		
0	173 (72.4)	178 (69.3)
1	51 (21.3)	50 (19.5)
2	15 (6.3)	29 (11.3)
Type of surgery, N (%)		
Colon resection	115 (48.1)	166 (64.6)
Low anterior resection + Hartmann^b^	83 (34.7)	50 (19.5)
Abdomino-perineal resection^c^	41 (17.2)	41 (16.0)
Neo-adjuvant treatment, *N* (% )		
(Chemo) radiotherapy^b^	99 (79.8)	73 (81.1)
Chemotherapy only	1 (0.4)	6 (2.3)

All percentages are given as percentage of number of patients within group for no anaemia/anaemia unless stated otherwise

^a^Median age patients with colon cancer 71 years, median age patients with rectal cancer 65 years

^b^Only patients with rectal cancers

^c^Including one patient with colon cancer in the anaemic group

**Table 2:** Patient characteristics of 496 patients undergoing curative abdominal resections for stage I–III colorectal cancer

Patient characteristics	No transfusion	Transfusion
Subjects, *N* (% of total)	297 (59.9)	199 (40.1)
Female, *N* (%)	136 (45.8)	98 (49.2)
Median age (year) at diagnosis^a^ (IQR)	66 (61–73)	73 (63–80)
Number CRC, no. (%)		
Colon	173 (58.2)	109 (54.8)
Rectum	124 (41.8)	90 (45.2)
ASA, *N* (%)		
1	70 (23.6)	10 (5.0)
2	149 (50.2)	96 (48.2)
3	70 (23.6)	78 (39.2)
4	8 (2.7)	15 (7.5)
pT stage, *N* (%)		
1	43 (14.5)	14 (7.0)
2	67 (22.6)	26 (13.1)
3	155 (52.2)	108 (54.3)
4	32 (10.8)	51 (25.6)
pN stage, *N* (%)		
0	213 (71.7)	138 (69.3)
1	59 (19.9)	42 (21.1)
2	25 (8.4)	19 (9.5)
Type of surgery, *N* (%)		
Colon resection	173 (58.2)	108 (54.3)
Low anterior resection + Hartmann^b^	82 (27.6)	51 (25.6)
Abdomino-perineal resection^c^	42 (14.1)	40 (20.1)
Neo-adjuvant treatment, *N* (% )		
(Chemo) radiotherapy^b^	97 (78.2)	75 (83.3)
Chemotherapy only	3 (1.0)	4 (2.0)

All percentages are given as percentage of number of patients within group (not transfused/transfused within 24 h before or after surgery) unless stated otherwise

^a^Median age patients with colon cancer 71 years, median age patients with rectal cancer 65 years

^b^Only patients with rectal cancers

^c^Including one patient with colon cancer for the transfused patients

Colon cancer, more advanced ASA class and pT Stage were all significantly associated with anaemia (*p* < 0.001) whereas gender (*p* = 0.69) and pN stage (*p* = 0.14) were not significantly associated with anaemia.

**Fig. 1:**
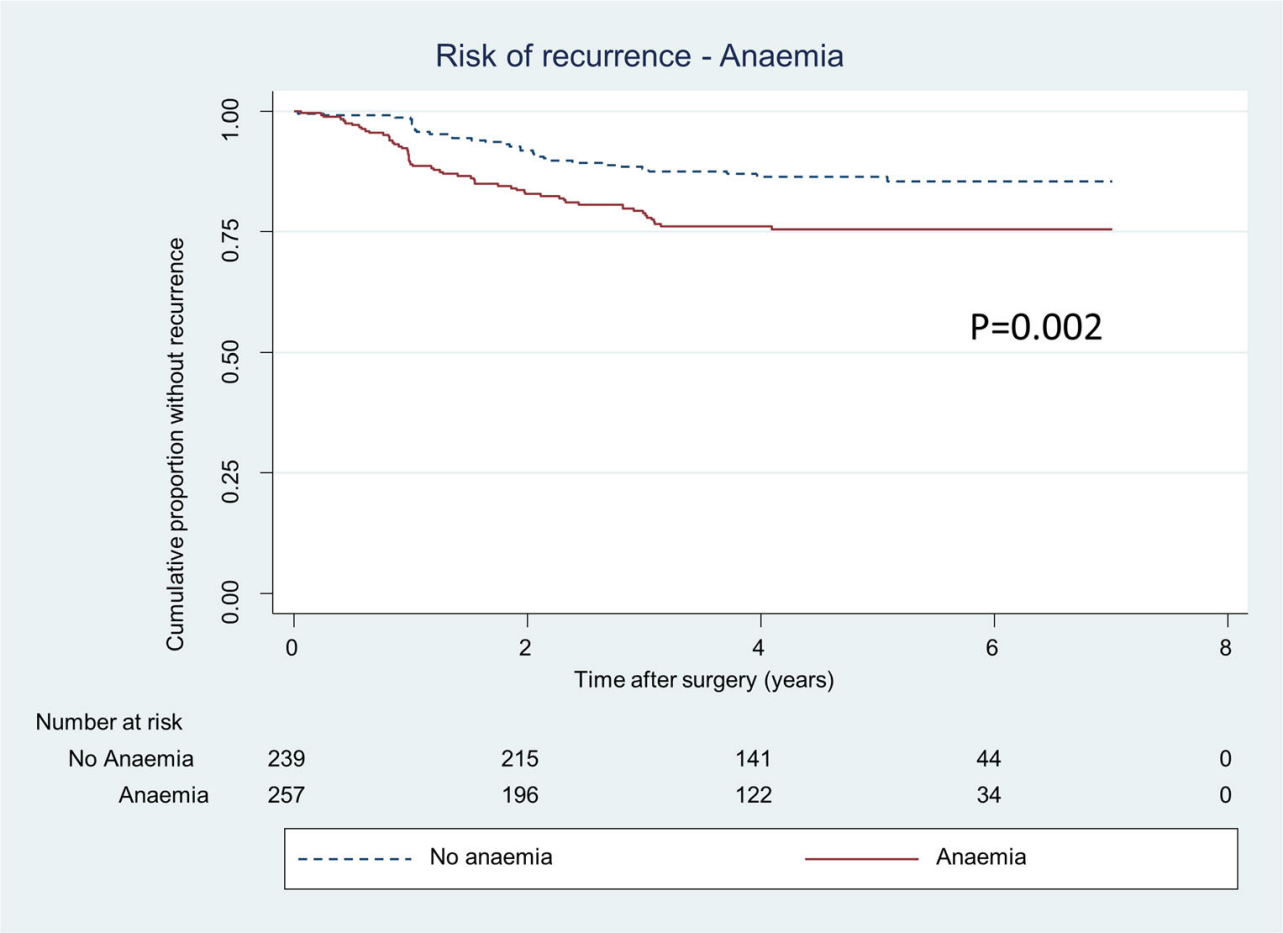
Proportion of patients, operated for CRC stages I–III, without recurrence divided in patients with preoperative anaemia and no anaemia. *p value* represents log-rank test

**Fig. 2:**
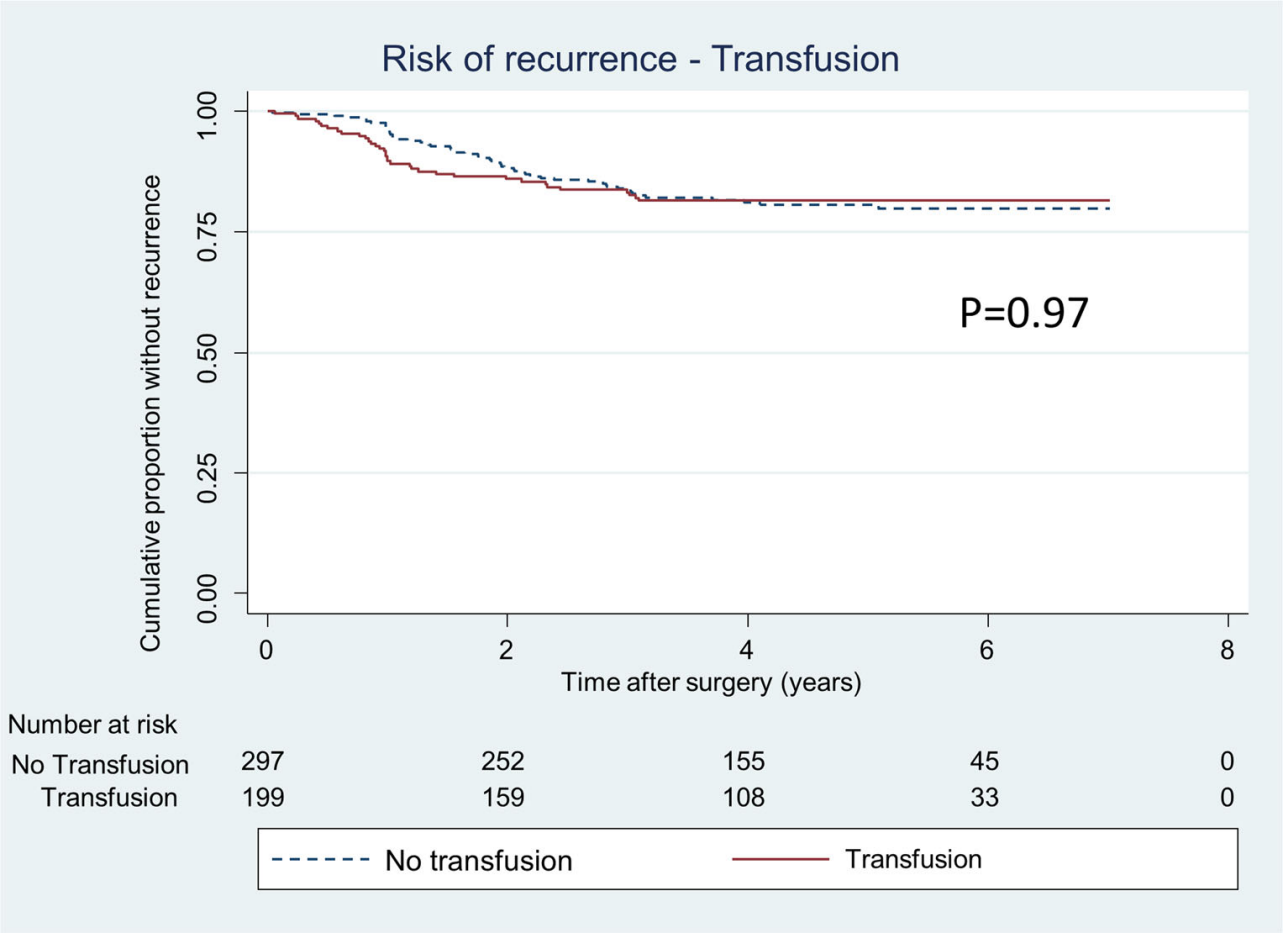
Proportion of patients, operated for CRC stages I–III, without recurrence divided in patients receiving blood transfusion within a day before or after surgery or not. *p value* represents log-rank test

The log-rank test analysis revealed that patients suffering from anaemia prior to surgery had a significantly increased risk for recurrence (*p* = 0.002) (Fig. 1), while the multivariate model with anaemia as dependent variable was not significant (HR 1.6, 95% CI 0.97–2.6). There was no association between receiving blood transfusion within 24 h of surgery and risk for recurrent disease (HR 0.7, 95% CI, 0.4–1.3) (Table 3, Fig. 2). Multivariate Cox regression analysis was also conducted for patients receiving a transfusion within 30 days of surgery (HR 0.7, 95% CI 0.4–1.2).

**Table 3:** Multivariate Cox regression analysis for risk of recurrence of 496 patients undergoing curative abdominal resections for stages I–III colorectal cancer

		Preoperative anaemia	Transfusion (± 1 day)	Preoperative anaemia and transfusion
	Events/person-years	Hazard ratio (95% CI)	Hazard ratio (95% CI)	Hazard ratio (95% CI)
Anaemia				
Yes	58/953.8	1.6 (0.97–2.6) ns		1.7 (1.1–2.9)*
No	32/1046.6	1.00 (ref)		1.00 (ref)
Transfusion				
Red blood cell	35/792.5		0.7 (0.4–1.3) ns	0.6 (0.4–1.1) ns
No transfusion	55/1207.9		1.00 (ref)	1.00 (ref)
Sex				
Female	39/983.8	0.8 (0.5–1.2) ns	0.8 (0.5–1.3) ns	0.8 (0.5–1.4) ns
Male	51/1016.6	1.00 (ref)	1.00 (ref)	1.00 (ref)
ASA grade				
1	20/329.5	1.00 (ref)	1.00 (ref)	1.00 (ref)
2	43/1052.3	0.9 (0.5–1.6) ns	0.9 (0.5–1.8) ns	0.9 (0.5–1.8) ns
3	23/539.1	0.7 (0.3–1.5) ns	0.9 (0.4–1.8) ns	0.8 (0.4–1.8) ns
4	4/79.6	1.4 (0.4–4.6) ns	1.8 (0.5–6) ns	1.8 (0.5–6.1) ns
pT				
1	2/264.7	1.00 (ref)	1.00 (ref)	1.00 (ref)
2	7/388.8	1.7 (0.3–8.3) ns	1.6 (0.3–8) ns	1.6 (0.3–7.9) ns
3	49/1054.7	4.9 (1.2–20.6)*	5.4 (1.3–22.6)*	4.7 (1.1–19.9)*
4	32/292.2	12.3 (2.8–53.8)**	15.9 (3.7–68.1)***	12.3 (2.8–53.5)**
pN				
0	48/1466.1	1.00 (ref)	1.00 (ref)	1.00 (ref)
1	17/420.4	1.3 (0.7–2.3) ns	1.3 (0.7–2.3) ns	1.3 (0.8–2.4) ns
2	25/113.9	4.7 (2.8–8)***	4.8 (2.9–8.2)***	5 (3–8.5)***
Type of surgery				
Colon resection	38/1127.8	1.00 (ref)	1.00 (ref)	1.00 (ref)
Low anterior resection + Hartmann	31/556.1	2.1 (0.9–5) ns	1.9 (0.8–4.6) ns	2 (0.8–4.9) ns
Abdomino-perineal resection	21/316.4	2.3 (0.9–6.1) ns	2.2 (0.8–6.1) ns	2.1 (0.8–5.8) ns
Neo-adjuvant treatment				
Yes	46/714.5	1.4 (0.6–3.1) ns	1.4 (0.6–3.1) ns	1.4 (0.6–3.2) ns
No	44/1285.9	1.00 (ref)	1.00 (ref)	1.00 (ref)

Analyses adjusted for age and blood loss as restricted cubic splines, 3 df

*ns* not significant, *ref* reference

*p < 0.05; **p < 0.01; ***p < 0.001

**Fig. 3:**
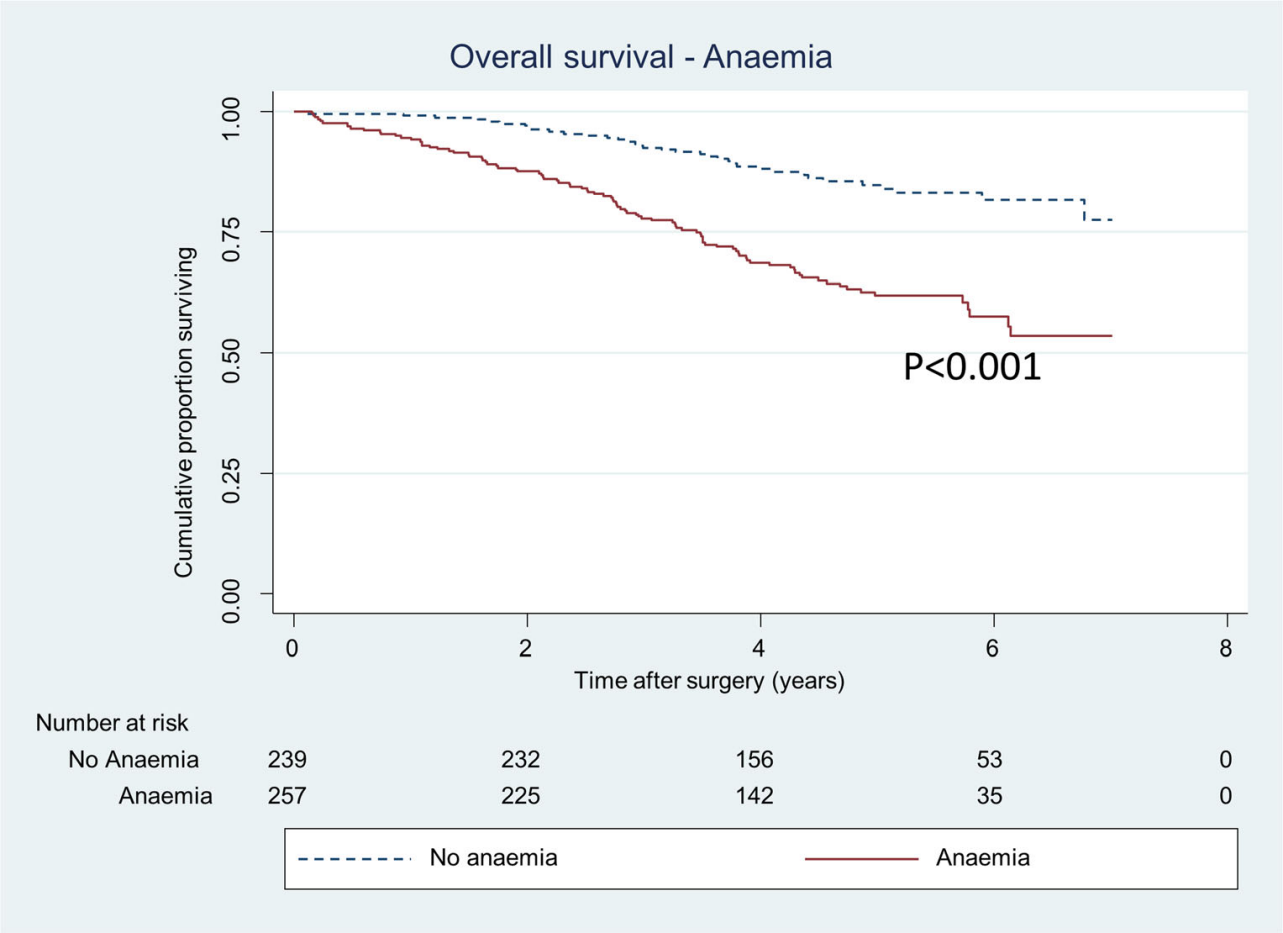
Proportion of patients, operated for CRC stages I–III, alive divided in patients with preoperative anaemia and no anaemia. *p value* represents log-rank test

**Fig. 4:**
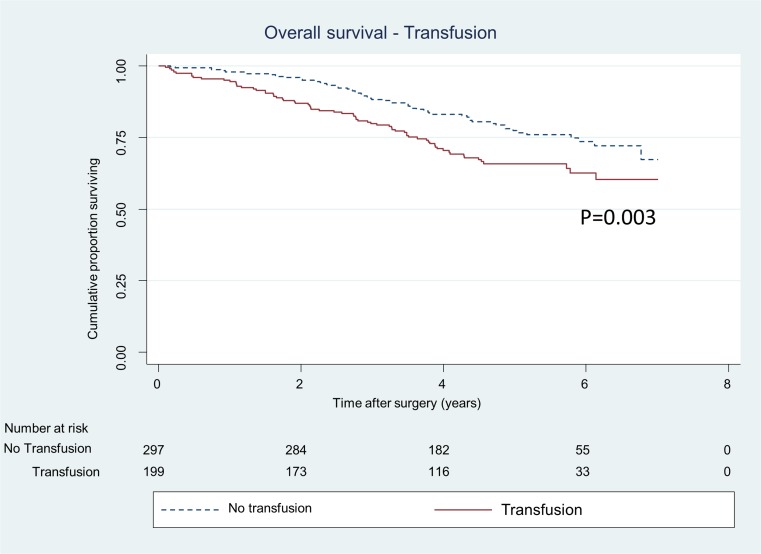
Proportion of patients, operated for CRC stages I–III, alive divided in patients receiving blood transfusion within a day before or after surgery or not. *p value* represents log-rank test

Anaemia prior to surgery was also associated with poorer overall survival (HR 2.1, 95% CI 1.4–3.2) (Table 4, Fig. 3). There was no association between blood transfusion and overall survival in the multivariate Cox regression analysis (HR 1.04, 95% CI 0.7–1.6) (Table 4) while the survival analysis tested with log-rank test was significant (Fig. 4).

**Table 4:** Multivariate Cox regression analysis for overall mortality of 496 patients undergoing curative abdominal resections for stage I–III colorectal cancer

		Preoperative anaemia	Transfusion (± 1 day)	Preoperative anaemia and transfusion
	Events/person-years	Hazard ratio (95% CI)	Hazard ratio (95% CI)	Hazard ratio (95% CI)
Anaemia				
Yes	95/1058.5	2.1 (1.4–3.2)**		2.3 (1.5–3.6)***
No	36/1129.4	1.00 (ref)		1.00 (ref)
Transfusion				
Red blood cell	67/848		1.04 (0.7–1.6) ns	0.8 (0.5–1.2) ns
No transfusion	64/1339.9		1.00 (ref)	1.00 (ref)
Sex				
Female	53/1066.9	0.6 (0.4–0.9)*	0.6 (0.4–0.9)**	0.6 (0.4–0.9)*
Male	78/1121	1.00 (ref)		1.00 (ref)
ASA grade				
1	11/382.3	1.00 (ref)	1.00 (ref)	1.00 (ref)
2	49/1147	1.1 (0.5–2.3) ns	1.2 (0.6–2.4) ns	1.1 (0.5–2.4) ns
3	57/576.8	1.9 (0.9–4.1) ns	2.2 (1–4.7)*	2 (0.96–4.4) ns
4	14/81.7	3.8 (1.6–9.2)**	4 (1.6–10)**	4.3 (1.7–10.6)**
pT				
1	5/270.2	1.00 (ref)	1.00 (ref)	1.00 (ref)
2	23/411.5	2.3 (0.8–6) ns	2.3 (0.9–6.2) ns	2.2 (0.8–6) ns
3	68/1163.5	1.4 (0.6–3.6) ns	1.8 (0.7–4.5) ns	1.4 (0.6–3.7) ns
4	35/342.7	1.8 (0.7–5) ns	2.5 (0.9–6.7) ns	1.9 (0.7–5.1) ns
pN				
0	86/1565.1	1.00 (ref)	1.00 (ref)	1.00 (ref)
1	20/463.8	0.8 (0.5–1.4) ns	0.9 (0.5–1.4) ns	0.8 (0.5–1.4) ns
2	25/159	3.6 (2.2–5.8)***	3.8 (2.3–6.1) ***	3.6 (2.2–5.9)***
Type of surgery				
Colon resection	84/1187.7	1.00 (ref)	1.00 (ref)	1.00 (ref)
Low anterior resection + Hartmann	24/642	0.6 (0.3–1.2) ns	0.5 (0.2–1.1) ns	0.5 (0.3–1.1) ns
Abdomino-perineal resection	23/358.2	0.8 (0.3–1.8) ns	0.7 (0.3–1.8) ns	0.7 (0.3–1.7) ns
Neo-adjuvant treatment				
Yes	40/820.8	1.4 (0.7–3) ns	1.4 (0.7–3) ns	1.5 (0.7–3.1) ns
No	91/1367.1	1.00 (ref)	1.00 (ref)	1.00 (ref)

Analyses adjusted for age and blood loss as restricted cubic splines, 3 df

*p < 0.05; **p < 0.01; ***p < 0.001

The analysis including both anaemia and transfusion as independent variables in the same model revealed an association between anaemia and risk for recurrence (HR 1.7, 95% CI 1.1–2.9) (Table 3), and death (HR 2.3, 95% CI 1.5–3.6) (Table 4). There was no association between transfusion and either of these outcomes (Table 3, Table 4).

## Discussion

Preoperative anaemia, present in approximately 50 % of the CRC patients in this study, was a strong predictor for impaired survival. There was no significantly increased risk of cancer recurrence in patients with preoperative anaemia. Blood transfusion was neither associated with increased risk for recurrence, nor with overall mortality in the present study.

This is a single centre retrospective cohort study using prospectively registered registry data. Consecutive patients undergoing radical abdominal resection for stages I–III CRC at the Karolinska University Hospital and living in the region were included. The minimum follow-up time in the study was 3 years. Karolinska University Hospital is one of the largest centres in Sweden, taking advanced cases from the Stockholm Region. Patients referred from other regions were not included in the trial since they are followed up at their nearest hospital.

For each patient, the lowest of all haemoglobin values registered within 2 months before the date of surgery was chosen for classification of anaemia. This was to minimise the risk of patients receiving preoperative iron medication or blood transfusion and being misclassified.

Data on blood transfusion was collected 2 months before surgery until 1 month after surgery. Analyses were performed for patients receiving blood transfusions within 1 day before or after surgery, since this time interval was considered the most interesting period with respect to impaired immunological response of the patient at the time of surgical trauma. One hundred ninety-nine patients (81 % of patients receiving blood transfusion) did so within this time interval. It can be argued that the immunological effect of blood transfusion in combination with surgical stress can be longer. Additional analyses for the period 1 month before or after surgery were performed regarding recurrent disease and survival.

The only available measure of co-morbidity was ASA class. This factor was adjusted for in the multivariate models since it was suspected to influence both survival and the risk for anaemia.

Abdomino-perineal resection is known to be associated with a higher risk for recurrence [21], hence the analyses were adjusted for surgical procedure. Although, such an association could not be shown in the current study. Analyses were also performed when dividing colonic resections into acute and elective procedures [22]. This did not affect the outcome estimates, and colon resections were thus analysed as one group.

The stage reported to the SCRCR is the stage derived from examination of the pathology specimen. Since 80 % of the patients with rectal cancer received neo-adjuvant (chemo) radiotherapy, and in total 36 % of all patients received any neo-adjuvant oncological treatment, down staging might have occurred in about a third of patients.

The risk for recurrence was associated with preoperative anaemia in the log-rank test but the confidence interval in the multivariate analysis did just include 1 (0.97–2.6) and was thus not significant. However, anaemia was significantly associated with the risk for recurrence in the model including both anaemia and transfusion. Therefore, this could be described as a trend and possibly this would have been significant in both models if the sample size was larger. More advanced T and N stage was associated with an increased risk for recurrence in the multivariate model. The association between anaemia and risk for recurrence has previously been shown in a recent study on stage II CRCs where anaemia was shown to be associated with a reduction in recurrence-free survival in T3N0M0 colon cancer patients only, but not in patients with T4N0M0 colon cancers [13]. In that study, adjustment was made for tumour but not for patient characteristics.

Blood transfusion, often defined in studies as given within 30 days before and after surgery, has repeatedly been found to be associated with an increased risk for recurrence [10]. Although there is little specific data on the mechanisms behind such an association, it has typically been attributed to the immunological side effects of blood transfusion [23]. The recurrence risk described previously was independent of tumour stage or when the transfusion was administered [10]. In the present study, no association was found between transfusion and risk for recurrence. The effect of transfusions administered within a day or 30 days before or after surgery showed practically identical results.

Anaemia prior to surgery, gender, ASA and pN-stage class were significantly associated with increased overall mortality regardless of tumour stage. The present study only included CRC patients stages I–III with curative abdominal resection, where all early postoperative deaths were excluded, making the cohort homogenous. Anaemia was associated with increased overall mortality in both the analysis for anaemia alone and in the combined analysis for anaemia and transfusion. The multivariate analyses revealed no association between transfusion and increased overall mortality although the log-rank test of the univariate Kaplan-Meyer model was significant.

Little is known about preoperative anaemia and the risk for death in patients operated for CRC [11–13]. Previous studies have shown an association between mild anaemia preoperatively and reduced overall survival 3–8 years after surgery in patients with CRC stages I–IV [12]. That study focused on the association between preoperative symptoms and survival [12]. Mild anaemia was associated with a more advanced stage but stage was not entered into the multivariate analyses [12]. The negative effect of preoperative anaemia on survival was also confirmed in another study including patients after both curative and palliative surgery [11].

Hazard ratios were virtually unaffected when analysing anaemia and transfusion as independent variables in the various multivariate models compared to HRs in the combined model although the association between anaemia and recurrence did not reach statistical significance in the model including only anaemia. Preoperative anaemia was, in both models, a significant risk factor for overall mortality. We were unable to show that transfusion has an impact on these outcomes in the multivariate models. It can be argued not to include both anaemia and blood transfusion in the same model since they are dependent of each other but, we believe, that showing both the separate models and the combined model increases the robustness of the results. The results of the present study stand in stark contrast to the conclusions of two meta-analyses where perioperative blood transfusion was considered to be consistently associated with risk for recurrence [10, 24]. One possible explanation could be that the Karolinska University hospital began using leucocyte-depleted blood components in 1997, so that all patients included in this study received these presumably less immune-modulatory blood components [23]. In addition, one must also consider the possibility of our study being under powered. While this may be indicated by the comparatively low statistical precision it must be kept in mind that the upper confidence interval for the estimate of the effect of blood transfusion on the risk for recurrence was 1.3. This would imply a lesser effect than that derived in the previous meta-analyses [10, 23].

## Conclusion

Preoperative anaemia, present in almost half of our CRC patients, was strongly associated with increased risk for overall mortality but not for recurrence. Blood transfusion was not associated with increased risk for recurrence or mortality.
